# Patterns of Signs That Telephone Crisis Support Workers Associate with Suicide Risk in Telephone Crisis Line Callers

**DOI:** 10.3390/ijerph15020235

**Published:** 2018-01-30

**Authors:** Tara Hunt, Coralie Wilson, Peter Caputi, Ian Wilson, Alan Woodward

**Affiliations:** 1 School of Medicine, University of Wollongong, Wollongong, NSW 2522, Australia; th719@uowmail.edu.au (T.H.); ianwil@uow.edu.au (I.W.); 2 Illawarra Health and Medical Research Institute, Wollongong, NSW 2522, Australia; 3 Centre for Mental Illness in Nowra District: Goals and Prevention (MINDtheGaP), Nowra, NSW 2541, Australia; Alan.Woodward@lifeline.org.au; 4 School of Psychology, University of Wollongong, Wollongong, NSW 2522, Australia; pcaputi@uow.edu.au; 5 Centre for Mental Health, University of Melbourne, Melbourne, VIC 3010, Australia; 6 Lifeline Research Foundation, Lifeline Australia, Canberra, ACT 2601, Australia; 7 Suicide Prevention Australia, Sydney, NSW 2000, Australia

**Keywords:** suicide, suicide intervention, telephone crisis-helpline, telephone crisis support, men, women, communication, suicide signs, suicide risk

## Abstract

Signs of suicide are commonly used in suicide intervention training to assist the identification of those at imminent risk for suicide. Signs of suicide may be particularly important to telephone crisis-line workers (TCWs), who have little background information to identify the presence of suicidality if the caller is unable or unwilling to express suicidal intent. Although signs of suicide are argued to be only meaningful as a pattern, there is a paucity of research that has examined whether TCWs use patterns of signs to decide whether a caller might be suicidal, and whether these are influenced by caller characteristics such as gender. The current study explored both possibilities. Data were collected using an online self-report survey in a Australian sample of 137 TCWs. Exploratory factor analysis uncovered three patterns of suicide signs that TCWs may use to identify if a caller might be at risk for suicide (mood, hopelessness, and anger), which were qualitatively different for male and female callers. These findings suggest that TCWs may recognise specific patterns of signs to identify suicide risk, which appear to be influenced to some extent by the callers’ inferred gender. Implications for the training of telephone crisis workers and others including mental-health and medical professionals, as well as and future research in suicide prevention are discussed.

## 1. Introduction

Telephone crisis lines play a pivotal role in suicide prevention systems by facilitating the identification of individuals with current thoughts of suicide and supporting them to seek ongoing mental-health care [1,2,3]. Emergent research suggests that the suicidal process is inherently dynamic with time-limited periods of acute intensity [4,5,6]. Crisis lines are one of the few intervention modalities that can respond to these short-term fluctuations by providing immediate support regardless of time of day or geographic location [7]. The individuals that staff telephone crisis lines, referred to as telephone crisis-line workers (TCWs), are usually trained personnel who provide crisis intervention remotely, on a once-off and time-limited basis [8,9,10]. In this time and information-limited telephone crisis-line context, TCWs are often reliant on the caller verbally expressing suicidality to identify callers with thoughts of suicide [11,12]. This context poses challenges for identifying suicidal callers [11]. Recent neuroscience research has found that suicidal ideation and behaviour indicate the presence of brain changes that physically impair the suicidal individual’s cognitive capability for identifying and describing emotions [13,14], which may contribute to a lack of disclosure about suicidal ideation [15,16]. If people who are imminently suicidal have impaired ability to articulate their suicidal ideation, the responsibility for preventing suicide is shifted to TCWs’ ability to accurately identify whether a caller might be suicidal from the information the caller can and does provide. 

### 1.1. Signs That Suicidality Might Be Present 

To identify whether a caller might be suicidal, TCWs often undergo training that includes learning signs of suicide, sometimes referred to as warning signs of suicide or suicide invitations. Suicide signs refer to indications of current suicidality that are *observed* by or *reported* to another, and indicate risk for suicide within minutes, hours or days, and are widely disseminated in a variety of psychoeducational and public mental health campaigns [17,18]. Widely accepted suicide signs include intense feelings of anger, increased alcohol or drug use, recklessness or engagement in risky activities, withdrawal from friends, family or society, agitation or restlessness, expresses that there is no reason for living, dramatic changes in mood, hopelessness, feeling trapped or stuck, inability to sleep or sleeps all the time, and intent to seek revenge [18]. TCWs are trained to be attentive to callers presenting with these signs. If suicide signs are detected the TCW is also trained to engage in direct questioning about the caller’s suicidal state before implementing procedures to ensure their safety. The importance of listening not just for veiled evidence of signs, but also correlates of the suicidal state suggesting that a caller might be suicidal, is highlighted by results of a recent systematic review which investigated the incidence of suicide communication prior to suicidal deaths [19]. The review found that across studies, approximately half of people who died by suicide did not directly state that they were suicidal [19]. Unlike direct expressions of intent to die by suicide, indirect expression of information that might suggest the caller is suicidal is open to interpretation [17,18].

### 1.2. The Role of Pattern Recognition in Identifying the Risk That a Caller Might Be Suicidal

The subjectivity involved in identifying whether an individual is at risk for suicide has led to the argument that information about the individual’s current state is only meaningful when interpreted as a pattern [17,18,20]. For example, one sign of suicide, such as social withdrawal, is likely to be insufficient to indicate to a TCW that the caller might be suicidal, but combined with talk of “leaving it all behind” and “feeling trapped”, is likely to trigger TCWs’ recognition that a caller might be suicidal. In this way, pattern recognition, defined as the “non-conscious recognition of problem-states based on patterns of features that prime appropriate scripts in memory” [21], is likely to play an important role in TCWs’ identification of suicidal callers. Importantly, pattern recognition is not a conscious or critical-thinking process [22]. Rather, it occurs when an individual subconsciously recognizes relationships between features of a presenting problem, which in turn, cues memories about the problem and strategies to manage it [23,24,25]. In the time and information limited telephone crisis support context, pattern recognition may be an effective strategy to identify callers at risk for suicide [26,27]. However, as noted, it is common practice across psychoeducational training for professionals and community-members to rote-learn single lists of signs rather than to learn how to recognize patterns of signs and correlates of suicide that suggest an individual might be suicidal, to determine appropriate intervention responses [28,29]. If TCWs decide how to respond to a caller on the basis of information patterns, it is likely these patterns are learned through informal learning that occurs via professional experience on telephone crisis lines, personal and lived experience, media reporting, print media, and television and film depictions [30,31].

### 1.3. The Influence of Callers’ Gender When Identifying Suicide Risk

Gender is a construct that refers to a system of social relations and practices that constitute people as two different categories, male or female [32,33], and defines the “differing characteristics of men and women and how they are *expected* to behave” [33] (p. 512, emphasis added). Although gender is a continuum rather than discrete categories of male/female, the dominant understanding of gender continues to be binary. In the context of suicide, ascribing suicide risk to binary categories of gender might be a consequence of reported population data [34]. For example, the overrepresentation of men in suicidal fatalities and women in suicide attempts [35,36,37], may have contributed to the perception that suicide is a masculine phenomenon [38,39,40,41]. Consequently, gender expectancies that are based on population data may inappropriately bias the interpretation of suicide signs at an individual level.

Research has found that suicide is often interpreted in ways that are consistent with social expectations of masculinity and femininity [39,42,43,44,45]. A study analyzing media coverage of suicide in Austria found that articles reporting female suicide deaths used more words related to mental-illness and socializing patterns than articles reporting male deaths, and articles reporting male suicide deaths used more words related to anger, spousal breakup and rejection than articles reporting female deaths [46]. These gendered patterns are also evident in friends’ and family members’ interpretation of suicidal presentation in suicide decedents [42,43]. In the context of telephone crisis support [11], gender is a discernible identity characteristic that is inferred, accurately or inaccurately, from vocal qualities such as timbre, pitch, resonance, and vocal style [47,48,49]. It is possible that the meaning TCWs associate with information provided by a caller is influenced by the caller’s gender. If this is accurate, it would suggest that gender may also influence TCWs’ recognition of information suggesting that a caller might be suicidal. 

### 1.4. The Influence of Telephone Crisis Workers’ Own Gender When Identifying Suicide Risk

The possibility that TCWs’ decision making might be influenced by caller gender extends to the TCWs’ own gender within the gender binary system. Expectancies about others’ behaviour are contained within one’s initial understanding of the other and self as male or female [33,50]. This perspective suggests that TCWs’ own gender and the expectancies associated with this identity might influence TCWs’ interpretation of patterns in the information provided by callers. A systematic review of the impact of gender on health-care communication found that the composition of gender-dyads of clinicians and patients had specific impacts on the quality and nature of health-care relationships and interactions [51]. Female patient-female doctor dyads appear to be characterised by psycho-social and biomedical talk with a person-centred approach [52], compared to male patient-female doctor dyads which involved interactions that exhibited low levels of technical language and the doctors’ use of a dominant tone [53]. In contrast, female patient-male doctor dyads involved a more interventionistic approach and were less patient-centred than with interactions in female-patient-female doctor dyads [53]; whereas, male patient-male doctor dyads had a biopsychosocial focus and friendly communication patterns [53,54]. Importantly, it appears that clinicians’ own gender can impact the style of health-care encounters that the clinician has with non-gender matched clients [52,53]. Accordingly, it is possible that the gender of the TCW interacts with the inferred gender of the caller to influence TCWs’ recognition of suicide risk. If the TCWs’ ability to identify whether a caller might be suicidal is influenced or confused by gender (either the caller’s or the TCW’s), TCWs may require additional training to ensure that their ability to accurately and effectively identify and support callers with thoughts of suicide is not compromised. 

### 1.5. The Current Study

The current study was exploratory and conducted to examine whether: (1) there might be patterns of suicide signs that TCWs use to decide whether a caller might be suicidal; and (2) if there are patterns, whether the gender of either the caller or TCW influences the suicide risk that TCWs associate with different patterns of signs. 

## 2. Methods

### 2.1. Participants 

A total of 148 active TCWs from a prominent Australian telephone crisis helpline consented to participate in the study. Eleven TCWs exited the survey before completion of the survey items in this study, and were excluded from the following analysis. A summary of the demographic details of participants can be found in Table 1. The sample is representative and reflects the crisis helplines male-female ratio. 

### 2.2. Design

An online survey with a measure assessing signs that TCWs associate with potential suicide risk was distributed to the national sample of TCWs from an Australian telephone crisis line. The development of the study was supported by a community-academic partnership (CAP) [55].

#### 2.2.1. Recruitment

**Figure 1:**
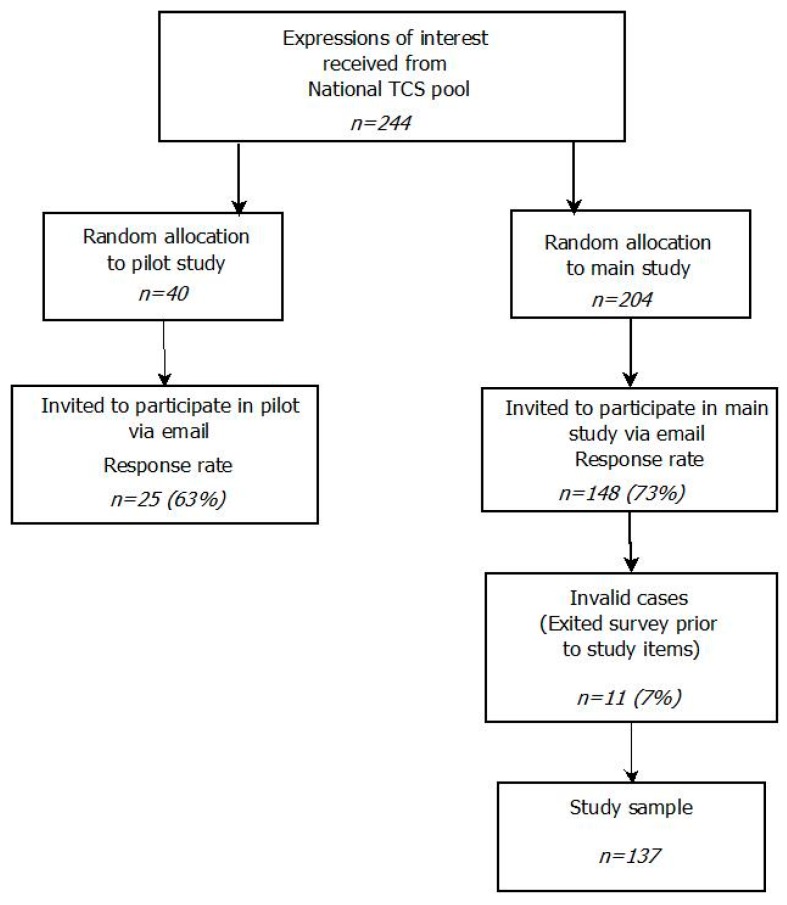
Participant recruitment flow chart.

The survey recruitment process and procedure were developed to make efficient use of TCWs’ time and workload. TCWs were only approached once to participate and were assigned to either the pilot or main study (see Figure 1). The study protocol was approved by the University of Wollongong Human Research Ethics Committee (16/135) and UnitingCare Queensland Human Research Ethics Committee (Wilson C.19016). After approval from relevant ethics review boards, crisis line centres that consented to participate in the study were asked to distribute an online expression of interest form among active TCWs. The TCWs who expressed interest in participating in the research project were randomly allocated to participate in either the pilot study, or the main study. Results from the pilot study fully supported conducting the main study as designed. Participants allocated to the main study were then invited to participate in the study by email. The response rate for the main study survey was 73% (*n* = 148). An independent survey administrator who was not a part of the research team communicated with participating TCWs. This assured the confidentiality and anonymity of research participants.

#### 2.2.2. Main Study Procedure

Participants were sent an electronic link to the online participant information sheet and study survey, which was hosted on SurveyMonkey^®^. The participant information sheet introduced the survey as “a study investigating how features of the crisis call influence how TCWs identify and respond to callers experiencing crisis”. If TCWs consented to participate in the study after reading the participant information sheet, they entered the survey to complete the first section that included demographic questions. After completing section one, participants were randomly allocated to an experimental condition (a male-female or female-male sign list) to counter balance potential order effects, and were asked to rate the suicide risk associated with signs of suicide. Two scales—one specified as for male callers and one specified for female callers—were randomized and presented on separate survey pages. Participants exited the survey after completing items in section two. 

### 2.3. Measures

*Demographics*: Participants responded to a series of demographic questions that assessed gender, age, home location, country of birth, years of experience as a TCW, and shift frequency and recency. TCWs also reported whether they had lived experience of suicide. 

*Risk associated with signs of suicide for male and female callers:* Participants read the instructions “The following scale will investigate the thoughts and behaviours expressed by a caller that would indicate to you they are at risk for suicide”, and were asked “Using the following scale, identify the behaviours that can be seen and thoughts that are expressed which would indicate that a (male/female) caller is at risk for suicide”. The scale comprised of a list of 11 common signs of suicide expressed by male or female callers: “expresses or behaves in ways which indicate intense feelings of anger”; “says they have increased alcohol or drug use”; “expresses or acts in ways which indicate recklessness or engagement in risky activities, seemingly without thinking”; “indicates they are withdrawing from friends, family, or society”; “expresses or behaves in ways which indicate agitation or restlessness”; “expresses there is no reason for living”; “expresses or behaves in a way which indicates dramatic changes in mood”; “says the future is hopeless”; “says they are feeling trapped, or stuck like there is no way out”; “expresses the inability to sleep, or says they sleep all the time” and “expresses the intent to seek revenge” [18,56]. Following recommendations from Ajzen [57,58], a seven-point Likert-type rating scale from 1 (*Low risk*) to 7 (*High risk*) was used to assess variance in participants’ responses. 

### 2.4. Data Screening and Analysis

Prior to analysis, scores for the risk associated with signs of suicide for male and female callers were examined in SPSS (IBM, Armonk, NY, USA), and parametric tests were deemed appropriate for use with the data. Exploratory Factor Analysis (EFA) is a statistical technique that is used to group together variables that are closely related [59] and was used to explore whether there were groups of signs that TCWs use to infer suicide risk, and whether these are different for male and female callers. Two EFA analyses were conducted; one included TCWs’ rating of suicide risk associated with signs of suicide in male callers (11 items), and the second included TCWs’ rating of suicide risk associated with signs of suicide in female callers (11 items). Both analyses were conducted using maximum likelihood extraction and direct oblimin rotation with a delta value of 0, following best practice recommendations [60,61]. The number of factors to be included in each EFA was determined by the Cattell’s scree test [62], which involves a visual inspection of the levelling-off of a plot of eigenvalues to determine the number of factors which account for the most variance. Each of the 137 participants in the study completed both suicide sign scales, which meant the minimum number of participants in both factor analyses was reached with an acceptable ratio of 12 cases for each variable [60]. For inclusion in the factor solution for each analysis the minimum loading requirement of 0.45 was selected, which represents a statistically significant correlation between the original variable and its factor at a power level of 80%, with an alpha level of 0.05 [63]. Items that loaded on more than one factor were removed if the cross-loading was greater than 0.4 [64]. The factor scores are a composite measure of each factor and were calculated for each participant using regression scores [63,65]. Higher factor scores indicated higher scores on scale items [63]. Bivariate correlations between the factor scores and age, gender, lived experience of suicide, and years of TCW experience were calculated, followed by a series of six hierarchical regression analyses to assess the extent to which TCWs’ own gender might influence the risk they associate with different patterns of signs for males and females. Regressions one to three were conducted in the sample of female TCSs and regressions four to six were conducted in the sample of male TCSs. In all regressions, factor scores were used as the dependent variable: regressions one and four used the scores for factor 1, regressions two and five used the factor scores for factor 2, and regressions three and six used the factor scores for factor 3. In all regressions, lived experience was controlled for and entered into the model at Step 1. TCWs’ gender was entered into each model at Step 2.

## 3. Results

To determine whether there are groups of signs that TCWs use to identify risk that a caller is suicidal, and whether these are different for male and female callers, two individual factor analyses were conducted on TCWs’ risk ratings for 11 suicide signs in male callers, and 11 suicide signs in female callers. For male callers, three factors were identified which explained 74% of total variance in TCWs’ risk rating. Nine of the 11 items that were entered into the EFA loaded on single factors with coefficients greater than 0.45. The item “withdrawal from friends, family, or society” cross-loaded onto Factors 1 and 2, and the item “increased alcohol or drug use” failed to reach the minimum loading required. Both items were both excluded from subsequent analysis. The total variance explained by the three factors was 47.22%, 17.97%, and 9.58%, respectively. Factor loadings are presented in Table 2. 

For female callers, there were also three factors that explained 73% of the total variance in TCWs’ risk rating. In contrast to the results for the male caller, for the female caller, 10 of the 11 items that were entered into the EFA loaded onto single factors with coefficients greater than 0.45, and are presented in Table 3. The item “Recklessness, or engagement in risky activities” failed to reach the minimum loading required and was excluded from subsequent analysis. The total variance explained by the three factors was 45.57%, 17.96%, and 9.61%, respectively.

For male and female callers the factors that were uncovered appear to be consistent with three broad patterns: mood; hopelessness; and anger. For each pattern, there were items that were in common for male and female callers. For the mood and anger categories, there were items that were different in male and female callers.

Common to male and female callers were items reflecting anger features (male callers: Factor 1; female callers: Factor 3): “intent to seek revenge” and “intense feelings of anger”; items reflecting mood features (male callers: Factor 2; female callers: Factor 1):“inability to sleep, or sleeps all the time”, ”agitation or restlessness”, and “dramatic changes in mood”; and items reflecting hopelessness features (male callers: Factor 3; female callers: Factor 2): “future is hopeless”, “feels trapped, or stuck like there is no way out”, and “no reason for living”. Differentiating risk recognized for male callers from female callers was one item in the anger pattern (male callers: Factor 1): “recklessness, or engagement in risky activities”; and differentiating risk recognized for female callers from male callers were two items in the mood pattern (female callers: Factor 1) “withdrawal from friends, family or society” and “increased alcohol or drug use”. Bivariate correlations were conducted to explore the relationships between demographic variables and the factor scores for each pattern for male and female callers. There were four small and significant associations: being a male TCW was correlated with higher risk ratings for the anger pattern among male callers (Factor 1), *r* = 0.18, *p* < 0.05, and higher risk ratings for the mood pattern among male callers (Factor 2), *r* = 0.21, *p* < 0.01; more years as a TCW was correlated with lower risk ratings for the mood pattern among male callers (Factor 2), *r* = −0.17, *p* < 0.05; and TCWs’ own lived experience of suicide was correlated with lower risk ratings for the mood pattern among female callers (Factor 1), *r* = −0.19, *p* < 0.05. As a precaution, TCWs’ lived experience was controlled for in the following regression analyses because over two-thirds of TCWs reported lived experience (see Table 1). 

Hierarchical regression was used to examine the extent to which TCWs’ gender was associated with the factor scores for male and female callers, with lived experience controlled for. The alpha level to determine statistical significance was set at *p* < 0.016 (calculated as *p* < 0.05/3) to adjust for family-wise error associated with multiple-comparisons. There was only one association that was close to significance across regressions: being a female TCW was associated with relating higher potential for suicide risk to hopelessness features among female callers at *p* = 0.018 (*b* = −0.40, Standard Error = 0.17, sr^2^ = −0.20; Adj *R*^2^ = 0.03, *F*(2, 133) = 3.06, *p* = 0.05).

## 4. Discussion

This study explored whether there were patterns of signs that TCWs use to recognize that a caller might be at risk for suicide, and whether these are different for male and female callers. Exploratory factor analysis uncovered three factors that reflected patterns of signs TCWs may use to identify whether a male or a female caller might be at risk for suicide. The patterns appear to reflect descriptions of mood, hopelessness, and anger. Importantly, while most signs that TCWs recognize in patterns were common for male and female callers, there were differences in specific suicide signs that TCWs related to the anger pattern in male callers and the mood pattern for female callers. This suggests that while TCWs may listen for common general patterns when deciding whether a male or female caller might be suicidal, the specific features of the anger and mood patterns may differ slightly depending on the inferred gender of the caller. Regression analyses found no significant evidence to suggest that TCWs’ own gender influences pattern recognition. 

TCWs are not trained in patterns to identify suicide risk, which suggests the finding of common patterns of signs used by TCWs to identify callers at risk for suicide may be an adaptive strategy to facilitate the identification of suicide risk in callers. In the time and information restricted context of telephone crisis support, TCWs’ ability to recognize suicidal potential within callers from patterns of anger, hopelessness, and mood signs may be particularly important if the caller has not directly and verbally expressed suicidal intent. Further, the subtle differences between male and female callers in the anger and mood pattern suggest that the patterns TCWs use to identify suicide risk in callers are not static, but may be changeable and reflective of the context of the caller. This finding suggests this sample of TCWs were not utilizing blank templates of characteristics ascribed to suicide risk, and appears to reflect the current movement in training away from risk categorisation and towards individualized approaches of suicide intervention [66,67]. The findings suggest that training which emphasizes individualized approaches to suicide intervention for all TCWs and face-to-face responders is an important step to improving the identification of suicidal individuals across disciplines and professions.

The possible utility of pattern recognition for identifying suicide risk in callers must also be tempered by the potential for pattern recognition to contribute to “suicidal candidacy” [68]. Suicide candidacy refers to theories or representations about the type of person who is expected to experience suicidality, e.g., the belief that suicidal individuals are socially withdrawn, depressed, and have experienced recent stressful life events. The results of the current study suggest that gender may also inform expectations about the characteristics of individuals at risk for suicide. Currently, there is no evidence to suggest that male and female callers to telephone helplines present with different risk profiles [2]. The finding that TCWs associated different anger signs with male suicide risk, and mood disruption signs with female suicide risk may reflect vulnerability to confirmation bias, within which, TCWs actively listen for these signs in caller presentation to determine whether a caller is suicidal. As a consequence, callers at risk for suicide may not be identified if they present differently to expectations. As pattern recognition is largely unconscious, the current findings point to the need for suicide intervention training across disciplines and professions to feature critical thinking and cognitive debiasing skills to ensure underlying interpretation biases do not impact the identification of suicide risk [69,70,71].

The patterns used by TCWs to identify the presence of risk for suicide appear to have little influence from their own gender or lived experience, which suggests that other factors are likely to have a role in developing TCWs’ understanding or recognition of specific patterns, e.g., experience on phones, suicide narratives in media etc. Motivations for volunteering and perspectives on helping are often assessed prior to acceptance in training programs to ensure TCWs are positioned to provide empathetic support and unconditional positive regard to callers which is not influenced by their own personal, emotional, or psychological needs. The current results suggest that, in the current sample, TCW screening and training has been effective at reducing the impact of TCW’s experience of their own gender on the help they provide to others. Findings from this study point to the need for training to continue to focus on supporting TCWs to provide flexible and adaptive interventions that are tailored to individual need, and uninfluenced by TCWs’ previous experiences.

The findings should be considered in light of limitations. Self-report data was collected from volunteer participants at one national telephone crisis helpline. Although the sample was representative and reflected the crisis-line’s male-female ratio, the sample size was small. Future research should be conducted to determine whether the findings can be generalised to other crisis helplines and organisations. Patterns of signs associated with suicide risk were assessed using commonly disseminated suicide warning signs [18,56]. These signs may not be exhaustive. In practice, TCWs are in vocal contact with the caller where there is reciprocal influence that can substantially influence the suicide intervention process. Consequently, there may be other unknown information sources that TCWs attend to when making decisions about suicidal callers. These might include cultural background, age grouping, and descriptions of co-occurring mental health issues. It may also be important to consider whether the current results are explained by the influence of underlying gender biases which may account for the differences in patterns of signs that are used to identify risk in male and female callers. Future research should address TCWs’ interpretation of, and response to, suicide callers in vivo to examine how gender may impact decision making with actual suicidal callers. If TCWs look for subtle differences in signs to identify male and female callers at risk for suicide, it is important to know the extent to which this might impact subsequent responses, including recognizing the need for referral, search and rescue operations, and selection of support strategies to alleviate distress and enhance coping. Namely, it needs to be established whether automatic and subconscious pattern recognition processes enhance or compromise the identification of and response to suicidal callers, and under what conditions.

## 5. Conclusions

This study is the first known to have explored whether TCWs use patterns of suicide signs to identify the presence of suicide risk in callers, and whether this is influenced by gender. The results suggest that TCWs may recognize patterns of signs that indicate a caller could be suicidal, and these are subtly and qualitatively different for male and female callers. Furthermore, the patterns recognized by TCWs did not appear to be influenced by TCWs’ own gender. Combined, the findings suggest that helpers trained in suicide intervention may use pattern recognition to identify potential suicide risk if a person does not explicitly state suicidal intent, and these patterns may be influenced by characteristics such as inferred gender. As much is at stake in suicide prevention, it is vital that the conditions under which pattern recognition is an effective component of the support process are thoroughly understood. As pattern recognition is by definition subconscious, the current results suggest that training practices must continue to support TCWs’ in treating each caller as separate and unique to provide empathetic and flexible suicide intervention that is suited to each caller’s needs. Future research should investigate whether the subtle differences in patterns of signs that TCWs use to identify suicide risk in male and female callers impacts their subsequent responses to callers they suspect might be suicidal. 

## Figures and Tables

**Table 1:** Demographics of Participants split by female (*n* = 98) and male (*n* = 39) telephone crisis-line workers (TCWs).

Demographics	Range	Female		Male		Total	
		*n*	*%*	*n*	*%*	*n*	*%*
Age	>25	10	10.2	1.0	2.6	11.0	8.0
	26–35	11	11.2	0.0	0.0	11.0	8.0
	36–45	20	20.4	4.0	10.3	24.0	17.5
	46–55	26	26.5	8.0	20.5	34.0	24.8
	56–65	21	21.4	18.0	46.2	39.0	28.5
	66<	10	10.2	8.0	20.5	18.0	13.1
Home Location	Metropolitan	61	62.2	19	48.7	80	58.4
	Regional	30	30.6	15	38.5	45	32.8
	Rural/Remote	7	7.1	5	12.8	12	8.8
Lived experience of suicide	Yes	71	72.4	27	69.2	98	71.5
	No	26	26.5	12	30.8	38	27.7
	Prefer not to respond	1	1.0	0	0.0	1	0.7
Lifeline position	Volunteer	85	86.7	31	79.5	116	84.7
	Employee	13	13.3	8	20.5	21	15.3
Years as a TCS	0–2 years	52	53.1	14	35.9	66	48.2
	3–5 years	24	24.5	10	25.6	34	24.8
	6–8 years	9	9.2	6	15.4	15	10.9
	9–11 years	6	6.1	4	10.3	10	7.3
	12–14 years	4	4.1	4	10.3	8	5.8
	15 years or longer	3	3.1	1	2.6	4	2.9
Shift frequency	More than once per week	9	9.2	6	15.4	15	10.9
	Once per week	38	38.8	21	53.8	59	43.1
	Once per fortnight	46	46.9	12	30.8	58	42.3
	Once per month	4	4.1	0	0.0	4	2.9
	Less than once per month	1	1.0	0	0.0	1	0.7
Shift recency	Less than 1 week ago	44	44.9	21	53.8	65	47.4
	1 week ago	22	22.4	6	15.4	28	20.4
	2 weeks ago	15	15.3	7	17.9	22	16.1
	3 weeks ago	7	7.1	1	2.6	8	5.8
	4 weeks ago	3	3.1	3	7.7	6	4.4
	More than 4 weeks ago	7	7.1	1	2.6	8	5.8

**Table 2:** Direct oblimin rotated factor structure of patterns of suicide signs associated with suicide risk in male crisis line callers.

Suicide Signs	Loadings		
	1	2	3
Intent to seek revenge	1.03		
Intense feelings of anger	0.56		
Recklessness, or engagement in risky activities	0.45		
Inability to sleep, or sleeps all the time		0.99	
Agitation or restlessness		0.80	
Dramatic changes in mood		0.59	
Future is hopeless			0.86
No reason for living			0.69
Feels trapped, or stuck like there is no way out			0.67
Cronbach’s alpha	0.79	0.87	0.74

**Table 3:** Direct oblimin rotated factor structure of patterns of suicide signs associated with suicide risk in female crisis line callers.

Suicide Signs	Loadings		
	1	2	3
Agitation or restlessness	0.83		
Dramatic changes in mood	0.78		
Inability to sleep, or sleeps all the time	0.78		
Withdrawing from friends, family or society	0.64		
Increased alcohol or drug use	0.60		
Future is hopeless		0.83	
No reason for living		0.66	
Feels trapped, or stuck like there is no way out		0.61	
Intense feelings of anger			0.80
Intent to seek revenge			0.76
Cronbach’s alpha	0.83	0.73	0.79
